# Bone mineral density, cervical spine degeneration, head and neck posture, and neck pain in the post-menopausal females: A pilot study

**DOI:** 10.1371/journal.pone.0257735

**Published:** 2021-09-20

**Authors:** Seok Woo Hong, Ki Tae Park, Yoon-Sok Chung, Yong Jun Choi, Jeong-Hyun Kang

**Affiliations:** 1 Department of Orthopedic Surgery, Kangbuk Samsung Hospital, Sungkyunkwan University School of Medicine, Seoul, Korea (ROK); 2 Department of Endocrinology and Metabolism, Ajou University School of Medicine, Suwon, Gyeonggi-do, Korea (ROK); 3 Clinic of Oral Medicine and Orofacial Pain, Institute of Oral Health Science, Ajou University School of Medicine, Suwon, Gyeonggi-do, Korea (ROK); Istanbul University Istanbul Faculty of Medicine: Istanbul Universitesi Istanbul Tip Fakultesi, TURKEY

## Abstract

The purpose of the present study was to reveal the relationship between degenerative changes in the cervical spine, head and neck postures, neck pain, and bone mineral density (BMD) of the total hip, femoral neck, and lumbar spine in post-menopausal females. In total, 116 females (mean age 60.4 ± 7.1 years; age range 50–80 years) were included. Participants were classified into three groups based on the T-score criteria of the total hip, femoral neck, and lumbar spine set by World Health Organization, respectively. The degree of neck pain was assessed using self-administered questionnaire, the Neck Disability Index. Cervical spine degeneration and head and neck postures were identified using the lateral cephalograms. Grading system for cervical degeneration included three categories of the radiographic alterations including disc height loss, osteophyte formation, and diffuse sclerosis. The areal BMD of the total hip, femoral neck, and lumbar spine were determined using dual-energy x-ray absorptiometry. Females with lower BMD exhibited lesser degree of neck pain and forward head posture (FHP) compared to those with normal BMD. Higher BMD seemed to be associated with more notable loss of the disc height at the level of C4-5. More prominent degenerative changes in the cervical spine were associated with higher areal BMD of the hip, femoral neck, and lumbar spine, altered head posture, and development of neck pain.

## Introduction

Aging accompanies with decreased bone mineral density (BMD) and degenerative changes in spinal structures, which can both affect the quality of life. Degeneration in the spine includes sclerosis of the vertebral body, osteophyte formation in the facet areas, and intervertebral disc space narrowing. Accelerated bone loss in the spinal structures could result in increased risk of incidental spinal fracture and altered spinal structures from degeneration could lead to abnormal neck function and pain in elder population [1,2].

The relationship among the head and neck postures, degenerative changes in the cervical spine, and neck pain in the elderly have been previously discussed [3]. Reduced paraspinal muscle volume due to aging could result in decreased stabilization of the vertebral spine and intervertebral disc [1,2]. This can lead to an abnormal force transmission across the facet joint and associated musculature, leading to the occurrence of cervical spine degeneration and mechanical hyperalgesia [4]. Increased neck pain can have a role in inducing a forward head posture (FHP), defined as holding the head out, anterior to the plane of the shoulders which serves as an antalgic posture for reducing neck pain by shortening the vertical extensor muscles [5,6]. In addition, FHP may also cause excessive capsular ligament stretch beyond biophysical limitations, thereby resulting in decreased pain threshold of the nerve endings and activation of proprioceptors in the facet joint capsules, which have an influence on development of the neck pain [7]. Hence, aging accelerates degenerative changes in the cervical spine leading to increased susceptibility of the neck pain and development of FHP in elderly.

Spontaneous bone loss is one of the main feature of aging and can cause increased risk of osteoporotic fracture and changes in vertebral structures such as excessive spinal curvatures [8]. Interestingly, the associations between decreased vertebral BMD and disc degenerations in the lumbar spine have been discussed previously, which reveals conflicting results. Several studies showed significant interactions between vertebral BMD and lumbar disc degeneration [9–12] that osteophytes, lumbar disc degeneration and compression could overestimate bone density interpretation [9,13]. Other suggested no significant association between hip BMD and degree of lumbar disc degeneration [13]. However, to the best of our knowledge, sparse reports have investigated the relationships among BMD, cervical spine degeneration, neck pain, and head and neck posture. One report proposed the significant relationship among the bone metabolism, cervical disc degeneration, and levels of the bone turnover markers and amino acids, while no information about the head and neck postures and neck pain were discussed [14]. Therefore, the purpose of the present study was to describe the relationship between degenerative changes in the cervical spine, head and neck postures, neck pain, and BMD of the lumbar spine and total hip in the elderly populations.

## Materials and methods

### Participants

This study was a retrospective cross-sectional study including the clinical and radiographic data of 116 post-menopausal female patients with temporomandibular disorders (TMDs) (mean age 60.4 ± 7.1 years; age range 50–80 years) who visited the TMDs·Orofacial Pain Clinic and Department of Endocrinology and Metabolism in a University Hospital from March, 2017 to November, 2020. The medical records of all subjects without missing data during this period were utilized. Females who underwent lateral cephalometric radiograph for diagnosis of TMD and had information about BMD measured by dual-energy x-ray absorptiometry (DEXA) of the total hip and lumbar spine within six months from the radiographic exam were included. Patients with following conditions were excluded from this study; history of treatment of spinal surgery or treatment in department of orthopedics, neurosurgery, and rehabilitation due to neck pain or low back pain; neurodegenerative disorders; craniofacial anomalies; autoimmune diseases; history of head and neck trauma prior to at least six months prior to study entry; history of spinal fracture; loss of posterior teeth; as well as wearing removable dentures.

We separately analyzed the associations between degree of the cervical spine degeneration, neck pain, head and neck posture, and BMD of the total hip, femoral neck, and lumbar spine, respectively. Participants were classified into three groups based on the T-score criteria of the total hip, femoral neck, and lumbar spine set by the World Health Organization (WHO). Several reports mentioned that the lumbar disc degeneration and height loss of the disc may cause distortion of the measurement of aBMD of the lumbar spine and suggested to adopt aBMD of the total hip or femoral neck for diagnosis of osteoporosis [9,13]. We adopted all three criteria for diagnosis of osteopenia and osteoporosis, including aBMD and T score of the total hip, femoral neck, and lumbar spine to reduce the measurement error and distortion. There were 49 females whose T scores from total hip were above -1.0 (T-score ≥ -1.0) (Normal-H). Sixty females whose T scores from total hip were between -1.0 to -2.5 (-2.5 < T-score <-1.0) were classified as Osteopenia-H and 7 participants whose T scores were equal or less than -2.5 (T-score ≤ -2.5) were as Osteoporosis-H. Whereas, 36 participants were classified as Normal-L, 53 as Osteopenia-L, and 27 as Osteoporosis-L based on T scores from the lumbar spine and 53 participants were classified as Normal-F, 57 as Osteopenia-F, and 6 as Osteoporosis-F based on T scores from the femoral neck.

Patients were diagnosed according to the Diagnostic Criteria for Temporomandibular Disorder (DC/TMD) Axis I [15]. The trigger points (TrPs) in the masticatory and cervical muscles were evaluated according to the criteria proposed by Simon and Travell [16]. The degree of subjective neck pain was determined using the self-administered questionnaire, the Neck Disability Index (NDI) at the time of taking the lateral cephalogram.

The research protocol was reviewed in compliance with the Helsinki Declaration and approved by the Institutional Review Board of the University Hospital (AJIRB-MDB-21-009). The Institutional Review Board committee approved a request to waive the documentation of informed consent due to the retrospective design of the study. All clinical data and personal information was blinded to all authors except corresponding author.

### Evaluation of TMD and trigger points in the masticatory and cervical muscles

TMD was diagnosed based on the DC/TMD axis I, supplemented by plain radiographs including orthopantomograms and lateral cephalograms [17,18]. Clinical parameters such as the degree of pain free opening and maximum unassisted opening as well as the duration of TMD symptoms including pain in the temple, jaw, and periauricular area; joint noise; and difficulties in opening and/or closing the mouth were assessed. A visual analog scale (VAS) based on the DC/TMD axis II was used to determine the extent of the subjective orofacial pain. Moreover, myofascial trigger points (TrPs) were bilaterally explored in the two masticatory muscles including temporalis and masseter muscles and four cervical muscles such as trapezius, sternocleidomastoid, sub-occipitalis, and splenius capitis muscles. TrPs were evaluated based on the criteria suggested by Simon and Travell [16].

### Measurement of BMD

The areal BMDs (aBMD, in grams per square centimeter) of the lumbar spine and total hip were determined using DEXA with Prodigy device (GE Lunar, Madison, WI, USA) and iDXA device (GE Lunar, Madison, WI, USA). We adopted the diagnostic criteria for osteoporosis by WHO. The criteria suggested by WHO did not include further information about the region of interest. Instead, International Society for Clinical Densitometry suggested to use total lumbar spine for spinal BMD measurement [19]. The coefficients of variation for BMD were 0.61% (total hip), 0.93% (femoral neck), and 0.87% (lumbar spine L1-L4).

### Appraising head and neck posture and degree of neck pain

**Fig 1:**
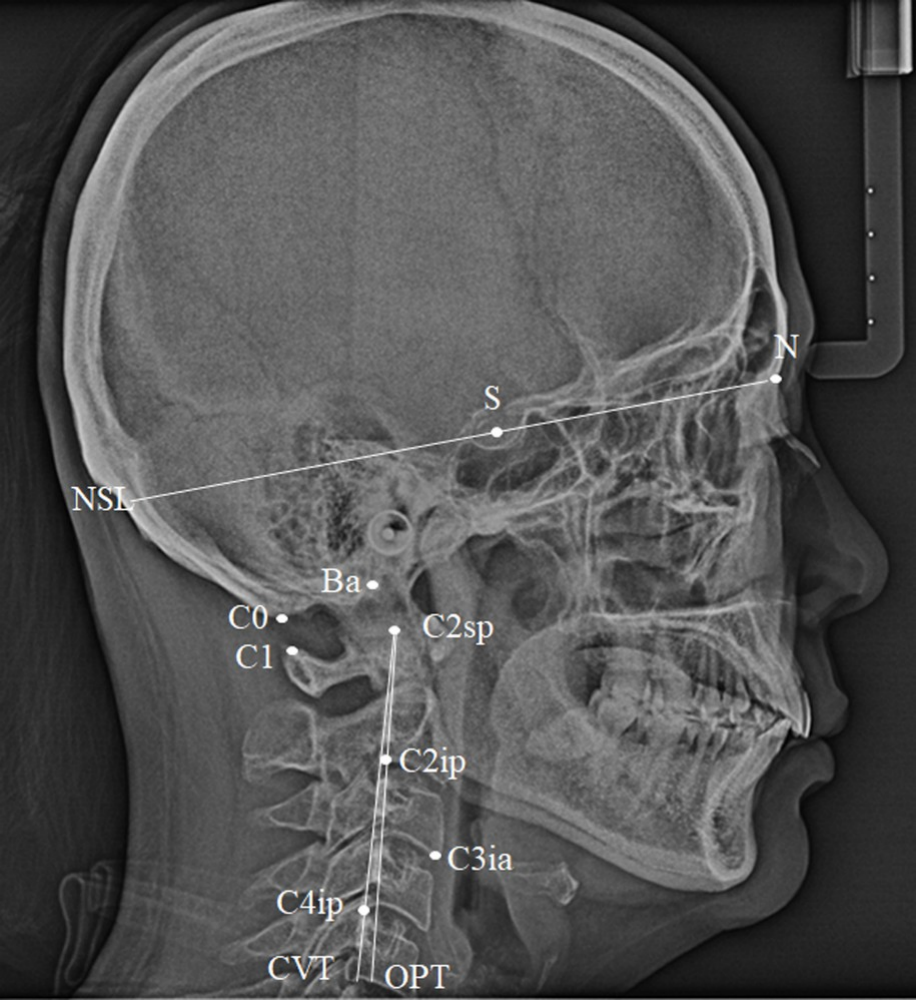
Cephalometric landmarks and variables used for analysis of the head and neck posture. Ba, basion; N. nasion; S, sella; C0. Base of the occiput; C1, the posterior arch of the atlas; C2, the spinous process of the second vertebra; C2sp, the most superior-posterior point on the body of the second vertebra; C2ip, the most inferior-posterior point on the body of the second vertebra; C3ia, the most inferior-anterior point on the body of the third vertebra; C4ip, the most inferior-posterior point on the body of the fourth vertebra; OPT, posterior tangent to the odontoid process through C2ip; CVT, posterior tangent to the odontoid process through inferior C4ip.

The amount of subjective neck pain was assessed using the NDI, self-administered questionnaire. One author (JHK) analyzed the craniofacial morphology and head and neck posture using lateral cephalogram using the V-ceph® 5.0 software (Cybermed, Seoul, Korea) from previous reports (Fig 1) [3,20–23]. All lateral cephlograms were obtained on the same radiographic machine in centric occlusion and the Frankfort horizontal plane parallel to the floor. The target-patient distance was 152.4 cm and the patient-film distance was 14 cm. One trained radiological technologist took all cephalograms. To assess inter-examiner reliability, one orofacial pain and TMD specialist (JHK) and one orthopedic surgeon (SWH) evaluated the ANB and OPT-CVT from 20 randomly selected cephalograms and the results from each examiner were compared (inter-examiner) using the intraclass correlation coefficient (ICC). ICC was 0.655, suggesting moderate agreement. Each examiner was blinded to the other. One observer (JHK) repeated the process after 2 weeks (intra-examiner) and data were compared using intraclass correlation coefficient (ICC). The ICC was 0.903 suggesting good agreement.

- SNA: angulation between the Sella-Nasion line and the Nasion-A point line
- SNB: angulation between the Sella-Nasion line and the Nasion-B point line
- ANB: calculated by subtracting SNB value from SNA value
- NSL-OPT: angulation between the Nasion-Sella line and line which connected odontoid process to the posterior-inferior point of the second vertebra [C2ip] (OPT)
- NSL-CVT: angulation between Nasion-Sella line and line which connected posterior tangent to the odontoid process to the posterior-inferior point of the fourth vertebra [C4ip] (CVT)
- OPT–CVT: angulation between the OPT and CVT
- Ba–C3ia: distance between the basion (Ba) and the most anterior-inferior point on the body of the third vertebra (C3ia)
- Cranium-atlas distance (C0–1): distance between the base of the occiput (C0) and the posterior arch of the atlas (C1)

### Determination of the degenerative changes in the cervical spine

This study adopted a grading system involving three major categories of the radiographic alterations of the cervical spine: “height loss”, “osteophyte formation”, and “diffuse sclerosis” [24]. “Total degeneration” was evaluated from sum of the three scores from each intervertebral space. No participants with ossification of posterior longitudinal ligament were included in this study.

**Fig 2:**
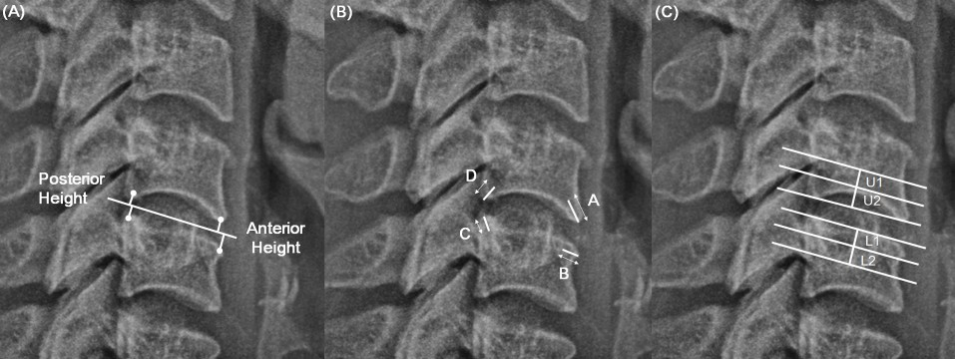
(A) “Height loss” was determined as the relative decrease of average anterior and posterior disc height compared to that before degeneration. The sum of the two anterior or two posterior disc heights was regarded as the actual anterior or posterior disc height, respectively (B) “Osteophyte formation” was defined as the sum of points assessed by the length of the osteophyte at the four edges. The length of the osteophyte was measured along the long axis from the border of the vertebral body to its ends (C) “Diffuse sclerosis” was evaluated as the sum of the points of both adjacent vertebral bodies. The upper half of the lower vertebral body (L1, L2) and lower half of the upper vertebral body (U1, U2) were divided into two regions.

“Height loss” was determined as the relative decrease of average anterior and posterior intervertebral disc height compared to that before degeneration. The individual disc height before degeneration was estimated by normal values suggested by Frobin [25]. For assessment, the actual anterior and posterior disc height was obtained. The distance of mid-plane of the disc to each of the four edges of the facet was measured. The sum of the two anterior or two posterior disc heights was regarded as the actual anterior or posterior disc height, respectively (Fig 2A). “Height loss” was graded as follows: 0 for no change, 1 for ≤ 33% change, 2 for ≥ 33% but < 66% change, and 3 for ≥ 66% change.

“Osteophyte formation” was defined as the sum of points assessed by the length of the osteophyte at the four edges. The length of the osteophyte was measured along the long axis from the border of the vertebral body to its ends (Fig 2B). The point system was as follows: 0 points for no osteophyte, 1 point for ≤ 2 mm length, 2 points for between 2–4 mm length, and 3 points for ≥ 4 mm length. “Osteophyte formation” was graded as follows: 0 for 0 points, 1 for 1–4 points, 2 for 5–8 points, and 3 for 9–12 points.

“Diffuse sclerosis” was evaluated as the sum of the points of both adjacent vertebral bodies. The upper half of the lower vertebral body (L1, L2) and lower half of the upper vertebral body (U1, U2) were divided into two regions. Those regions affected by sclerosis were regarded as the case of diffuse borders and the thickened bony endplate (Fig 2C). The point system applied was as follows: 0 points for no sclerosis, 1 point for less than half affected, and 2 points for more than half or completely affected. The grading system of “diffuse sclerosis” indicated 0 for 0 points, 1 for 1 point, 2 for 2 points, and 3 for 3–4 points.

“Total degeneration” was defined as the sum of the grades of “height loss”, “osteophyte formation”, and “diffuse sclerosis”. The grading system was as follows: grade 0 for 0 points (no degeneration), 1 (mild degeneration) for 1–3 points, 2 (moderate degeneration) for 4–6 points, and 3 (severe degeneration) for 7–9 points.

Determination of degree of the cervical degeneration was performed independently by two orthopedic surgeons (SWH and KTP). Cohen’s kappa test was used to assess the reliability and the coefficient was 0.755, suggesting good agreement. One observer (SWH) repeated the process one month later (intra-examiner) and and the coefficient was 0.753, suggesting good agreement. Each observer was blinded to the other and results from DEXA and clinical information including head and neck posture and degree of neck and orofacial pain, was blinded to each observer also.

### Statistical evaluation

The normality of data was affirmed using the Shapiro-Wilk normality test to adopt parametric statistical testing. To compare the differences in demographic features, aBMD and T-score of the total hip, femoral neck, and lumbar spine, clinical parameters, head and neck posture, and severity of degenerative changes of the cervical spine, and number of TrPs in the masticatory and cervical muscles among the groups, one-way analysis of variance followed by post hoc analysis with Bonferroni’s test and Chi-square test were used for continuous and categorical variables, respectively. The relationship between age, body mass index (BMI), BMD, cervical degeneration, head and neck postures, and degree of neck pain were determined using multivariate linear regression test. The dependent variables were aBMD of the total hip, femoral neck, and lumbar spine, respectively. The statistical analysis were performed using SPSS, version 25.

## Results

### Demographic features, clinical evaluation, and BMD

No significant differences of the age and parameters related with TMD such as durations of TMD symptoms, amount of pain free opening and maximum unassisted opening, VAS, and number of active and latent TrPs from masticatory and cervical muscles were observed among three groups which was classified based on T-scores from the total hip. Meanwhile, similar tendency was detected among the groups based on the T-scores from the femoral neck and lumbar spine. Degree of neck pain was higher in the Normal-H, Normal-F, and Normal-L groups compared to Osteoporosis-H, Osteoporosis-F, and Osteoporosis-L groups, respectively (Table 1).

**Table 1:** Demographic characteristics, bone mineral density, and features of TMD symptoms and neck pain in study population.

**Total hip**					
	Normal-H (n = 49)	Osteopenia-H (n = 60)	Osteoporosis-H (n = 7)	*P* value	Post-hoc analysis
Age	60.1 ± 8.3	61.3 ± 7.0	64.3 ± 7.3	0.067	
BMI	24.0 ± 3.1	23.5 ± 3.2	22.7 ± 2.5	0.056	
aBMD of total hip	0.99 ± 0.08	0.81 ± 0.06	0.67 ± 0.05	< 0.001**	Normal-Osteopenia, Normal-Osteoporosis, Osteopenia-Osteoporosis
T score of total hip	0.17 ± 0.68	-1.36 ± 0.48	-2.53 ± 0.43	< 0.001**	Normal-Osteopenia, Normal-Osteoporosis, Osteopenia-Osteoporosis
aBMD of femoral neck	0.91 ± 0.06	0.74 ± 0.05	0.63 ± 0.02	< 0.001**	Normal-Osteopenia, Normal-Osteoporosis, Osteopenia-Osteoporosis
T score of femoral neck	-0.25 ± 0.51	-1.61 ± 0.59	-2.63 ± 0.15	< 0.001**	Normal-Osteopenia, Normal-Osteoporosis, Osteopenia-Osteoporosis
aBMD of total lumbar spine	1.11 ± 0.17	0.95 ± 0.12	0.87 ± 0.18	< 0.001**	Normal-Osteopenia, Normal-Osteoporosis
T score of total lumbar spine	-0.39 ± 1.41	-1.67 ± 1.04	-2.34 ± 1.53	< 0.001**	Normal-Osteopenia, Normal-Osteoporosis
Duration of TMD symptoms (months)	20.8 ± 59.4	25.1 ± 41.3	18.9 ± 35.0	0.880	
Pain free opening (mm)	41.4 ± 8.7	41.7 ± 7.7	41.4 ± 7.7	0.982	
Maximum unassisted opening (mm)	73.4 ± 7.7	43.0 ± 7.2	42.0 ± 7.5	0.891	
VAS	4.73 ± 2.59	4.53 ± 2.45	5.86 ± 2.48	0.417	
NDI	16.0 ± 9.2	11.1 ± 5.6	11.8 ± 7.4	0.034*	Normal-Osteoporosis
Number of active TrPs in masticatory muscles†	0 (0–1)	0 (0–2)	0 (0–4)	0.470	
Number of active TrPs in cervical muscles†	0 (0–0)	0 (0–0)	0 (0–2)	0.080	
Number of latent TrPs in masticatory muscles†	0 (0–1)	0 (0–1)	0 (0–2)	0.345	
Number of latent TrPs in cervical muscles†	0 (0–0)	0 (0–0)	0 (0–0)	0.118	
**Femoral neck**					
	Normal-F (n = 53)	Osteopenia-F (n = 57)	Osteoporosis-F (n = 6)	*P* value	Post-hoc analysis
Age	60.8 ± 8.1	61.7 ± 6.9	65.2 ± 7.6	0.065	
BMI	23.9 ± 3.1	23.5 ± 3.3	21.1 ± 2.6	0.111	
aBMD of total hip	0.98 ± 0.09	0.81 ± 0.06	0.67 ± 0.06	< 0.001**	Normal-Osteopenia, Normal-Osteoporosis, Osteopenia-Osteoporosis
T score of total hip	0.05 ± 0.76	-1.39 ± 0.51	-2.53 ± 0.48	< 0.001**	Normal-Osteopenia, Normal-Osteoporosis, Osteopenia-Osteoporosis
aBMD of femoral neck	0.91 ± 0.07	0.74 ± 0.05	0.62 ± 0.01	< 0.001**	Normal-Osteopenia, Normal-Osteoporosis, Osteopenia-Osteoporosis
T score of femoral neck	-0.25 ± 0.56	-1.71 ± 0.43	-2.67 ± 0.12	< 0.001**	Normal-Osteopenia, Normal-Osteoporosis, Osteopenia-Osteoporosis
aBMD of total lumbar spine	1.10 ± 0.17	0.94 ± 0.13	0.91 ± 0.16	< 0.001**	Normal-Osteopenia, Normal-Osteoporosis
T score of total lumbar spine	-0.45 ± 1.38	-1.76 ± 1.10	-2.00 ± 1.35	< 0.001**	Normal-Osteopenia, Normal-Osteoporosis
Duration of TMD symptoms (months)	19.4 ± 57.3	26.3 ± 42.1	21.7 ± 37.5	0.769	
Pain free opening (mm)	40.9 ± 8.6	42.2 ± 7.5	41.2 ± 8.4	0.682	
Maximum unassisted opening (mm)	42.9 ± 7.7	43.5 ± 7.1	41.8 ± 8.2	0.843	
VAS	4.75 ± 2.54	4.54 ± 2.48	5.67 ± 2.66	0.570	
NDI	16.0 ± 10.1	11.4 ± 5.7	11.5 ± 7.1	0.271	Normal-Osteoporosis
Number of active TrPs in masticatory muscles†	0 (0–1)	0 (0–2)	1 (0–4)	0.361	
Number of active TrPs in cervical muscles†	0 (0–0)	0 (0–0)	1 (0–1.5)	0.101	
Number of latent TrPs in masticatory muscles†	0 (0–1.5)	0 (0–1)	0 (0–2)	0.292	
Number of latent TrPs in cervical muscles†	0 (0–0)	0 (0–0)	0 (0–1.5)	0.099	
**Lumbar spine**					
	Normal-L (n = 36)	Osteopenia-L (n = 53)	Osteoporosis-L (n = 27)	*P* value	Post-hoc analysis
Age	59.7 ± 8.7	59.6 ± 7.9	61.8 ± 6.3	0.456	
BMI	24.3 ± 3.2	23.6 ± 2.7	22.5 ± 3.8	0.083	
aBMD of total hip	0.97 ± 0.12	0.86 ± 0.09	0.78 ± 0.09	< 0.001*	Normal-Osteopenia, Normal-Osteoporosis, Osteopenia-Osteoporosis
T score of total hip	0.02 ± 1.02	-0.93 ± 0.73	-1.64 ± 0.77	< 0.001*	Normal-Osteopenia, Normal-Osteoporosis, Osteopenia-Osteoporosis
aBMD of femoral neck	0.89 ± 0.11	0.79 ± 0.09	0.72 ± 0.08	< 0.001*	Normal-Osteopenia, Normal-Osteoporosis, Osteopenia-Osteoporosis
T score of femoral neck	-0.40 ± 0.95	-1.23 ± 0.71	-1.82 ± 0.67	< 0.001*	Normal-Osteopenia, Normal-Osteoporosis, Osteopenia-Osteoporosis
aBMD of total lumbar spine	1.22 ± 0.11	0.98 ± 0.07	0.82 ± 0.08	< 0.001**	Normal-Osteopenia, Normal-Osteoporosis, Osteopenia-Osteoporosis
T score of total lumbar spine	0.54 ± 0.91	-1.44 ± 0.54	-2.74 ± 0.63	< 0.001**	Normal-Osteopenia, Normal-Osteoporosis, Osteopenia-Osteoporosis
Duration of TMD symptoms (months)	14.8 ± 28.0	33.0 ± 65.8	13.9 ± 24.5	0.127	
Pain free opening (mm)	42.9 ± 8.4	42.5 ± 6.7	40.0 ± 9.2	0.059	
Maximum unassisted opening (mm)	44.3 ± 8.1	43.7 ± 5.9	40.4 ± 8.5	0.087	
VAS	4.61 ± 2.49	4.57 ± 2.47	5.07 ± 2.66	0.675	
NDI	13.2 ± 7.2	9.20 ± 6.44	12.5 ± 6.1	0.040*	Normal-Osteopenia
Number of active TrPs in masticatory muscles†	0 (0–1)	0 (0–2)	0 (0–2)	0.470	
Number of active TrPs in cervical muscles†	0 (0–0)	0 (0–0)	0 (0–0)	0.080	
Number of latent TrPs in masticatory muscles†	0 (0–2)	0 (0–1)	0 (0–1)	0.345	
Number of latent TrPs in cervical muscles†	0 (0–0)	0 (0–0)	0 (0–0)	0.118	

BMI, body mass index; aBMD, areal bone mineral density; NDI, neck disability index; TMD, temporomandibular disorders; TrP, trigger point; VAS, visual analog scale.

Descriptive values are shown as mean ± SD.

†Descriptive values are shown as median (25–75% percentile).

Data obtained from one-way ANOVA.

^†^Data obtained from Chi-square test.

* P < 0.05,

** *P* < 0.001 by one-way ANOVA and Chi square test.

### Severity of degeneration in the upper cervical spine

Significant differences of degree of osteophyte formation at the level of C2-3 and C3-4 were detected among three groups from all criteria. The differences of amount of the disc height loss at the level of C2-3 and total grade of degeneration at the level of C4-5 showed significance only among the groups classified based on the T-scores from the total hip and femoral neck not from the lumbar spine (Table 2).

**Table 2:** Severity of degeneration of the cervical spine in study population.

**Total hip**				
	Normal-H (n = 49)	Osteopenia-H (n = 60)	Osteoporosis-H (n = 7)	*P* value
C2-3 height loss	2 (1–3)	2 (1–2)	1 (1–1)	0.015*
C2-3 osteophyte formation	2 (2–2)	2 (1–2)	1 (1–2)	0.014*
C2-3 sclerosis	2 (1–2)	2 (1–2)	1 (1–2)	0.018*
C2-3 total grade	2 (2–3)	2 (2–2)	1 (1–2)	0.002*
C3-4 height loss	2 (2–3)	2 (2–2)	2 (1–2)	0.147
C3-4 osteophyte formation	2 (2–2)	2 (1–2)	1 (1–2)	0.008*
C3-4 sclerosis	2 (2–3)	2 (2–3)	2 (1–2)	0.547
C3-4 total grade	2 (2–3)	2 (2–3)	2 (2–2)	0.018*
C4-5 height loss	2 (2–3)	2 (2–2)	2 (2–2)	0.040*
C4-5 osteophyte formation	2 (2–2)	2 (2–2)	2 (1–2)	0.125
C4-5 sclerosis	3 (2–3)	3 (2–3)	3 (2–3)	0.771
C4-5 total grade	3 (2–3)	2 (2–3)	2 (2–3)	0.016*
**Femoral neck**				
	Normal-F (n = 53)	Osteopenia-F (n = 57)	Osteoporosis-F (n = 6)	*P* value
C2-3 height loss	2 (1–3)	2 (1–2)	1 (1–1)	< 0.001**
C2-3 osteophyte formation	2 (2–2)	2 (1–2)	1 (1–2)	0.007*
C2-3 sclerosis	2 (1–2)	2 (1–2)	1 (1–2)	0.112
C2-3 total grade	2 (2–3)	2 (2–2)	1 (1–2)	< 0.001**
C3-4 height loss	2 (2–3)	2 (2–2)	2 (1–2)	0.096
C3-4 osteophyte formation	2 (2–2)	2 (1–2)	1 (1–2)	0.010*
C3-4 sclerosis	2 (2–3)	2 (2–3)	1.5 (1–2.25)	0.383
C3-4 total grade	2 (2–3)	2 (2–3)	2 (2–2)	0.034*
C4-5 height loss	2 (2–3)	2 (2–2)	2 (1.75–2)	0.090
C4-5 osteophyte formation	2 (2–2)	2 (2–2)	2 (1–2)	0.255
C4-5 sclerosis	3 (2–3)	3 (2–3)	2.5 (1.75–3)	0.743
C4-5 total grade	3 (2–3)	2 (2–3)	2 (2–3)	0.039*
**Lumbar spine**				
	Normal-L (n = 36)	Osteopenia-L (n = 53)	Osteoporosis-L (n = 27)	*P* value
C2-3 height loss	2 (1–3)	2 (1–2)	1.5 (1–2)	0.446
C2-3 osteophyte formation	2 (2–2)	2 (1–2)	2 (1–2)	0.040*
C2-3 sclerosis	2 (1–2)	2 (2–2.75)	2 (1–2)	0.217
C2-3 total grade	2 (2–3)	2 (1–2)	2 (2–2)	0.402
C3-4 height loss	2 (2–3)	2 (2–3)	2 (2–2)	0.414
C3-4 osteophyte formation	2 (1.25–2)	2 (2–2.75)	1.5 (1–2)	0.047*
C3-4 sclerosis	2 (2–3)	2 (1.25–3)	2 (2–2)	0.166
C3-4 total grade	2 (2–3)	2 (2–3)	2 (2–2)	0.018*
C4-5 height loss	2 (2–3)	2 (2–3)	2 (2–2)	0.033*
C4-5 osteophyte formation	2 (2–2)	2 (2–2)	2 (2–2)	0.453
C4-5 sclerosis	2 (2–3)	3 (2–3)	3 (2–3)	0.281
C4-5 total grade	3 (2–3)	3 (2–3)	2 (2–3)	0.226

C2, the second vertebra; C3, the third vertebra; C4, the forth vertebra; C5, the fifth vertebra; 1, mild degeneration; 2, moderate degeneration; 3, severe degeneration.

Descriptive values are shown as median (25–75% percentile).

Data obtained from Chi-Square test.

* P < 0.05,

** *P* < 0.001 by Chi-Square test.

### Head and neck postures and facial profiles

There were no statistical differences of facial profiles in three groups from all criteria. The females in the Normal-H, Normal-F, and Normal-L showed more severe FHP compared to females in other groups. A significantly higher values of OPT-CVT in Normal groups were found among three groups from all criteria. The results from Bonferroni’s post-hoc analysis demonstrated that values of the OPT-CVT in the Normal-H and Normal-F groups was significantly higher in those in the Osteopenia-H and Osteoporosis-H and Osteopenia-F and Osteoporosis-F, respectively. In addition, value of Ba-C3ai was also larger in the Normal-L group compared to in the Osteoporosis-L group (Table 3).

**Table 3:** Variables related with head and neck postures and facial profile.

**Total hip**					
	Normal-H (n = 49)	Osteopenia-H (n = 60)	Osteoporosis-H (n = 7)	*P* value	Post-hoc analysis
SNA (degree)	82.3 ±3.8	82.6 ± 3.8	81.4 ± 4.2	0.689	-
SNB (degree)	78.3 ± 3.6	78.8 ± 3.7	78.8 ± 3.5	0.776	-
ANB (degree)	4.09 ± 2.26	3.87 ± 2.41	2.87 ± 1.01	0.468	-
NSL-OPT (degree)	79.4 ± 7.0	78.3 ± 6.5	77.9 ± 6.1	0.645	-
NSL-CVT (degree)	73.5 ± 6.1	73.2 ± 6.4	75.1 ± 7.3	0.591	-
OPT-CVT (degree)	6.91 ± 2.35	5.13 ± 2.77	2.80 ± 1.45	0.003*	Normal-Osteopenia, Normal-Osteoporosis
Ba-C3ai (mm)	113.3 ± 8.6	112.3 ± 8.1	110.6 ± 9.1	0.662	-
C0-C1 (mm)	12.1 ± 6.8	13.9 ± 5.7	9.50 ± 5.40	0.117	-
**Femoral neck**					
	Normal-F (n = 53)	Osteopenia-F (n = 57)	Osteoporosis-F (n = 6)	*P* value	Post-hoc analysis
SNA (degree)	82.4 ± 3.7	82.6 ± 3.8	80.8 ± 4.2	0.545	
SNB (degree)	78.2 ± 3.5	78.9 ± 3.8	78.4 ± 3.8	0.658	
ANB (degree)	4.26 ± 2.37	3.73 ± 2.29	2.43 ± 4.21	0.170	
NSL-OPT (degree)	79.2 ± 6.9	78.6 ± 6.8	76.1 ± 4.3	0.533	
NSL-CVT (degree)	72.3 ± 6.0	73.6 ± 6.8	73.0 ± 5.3	0.560	
OPT-CVT (degree)	6.91 ± 2.26	4.93 ± 2.84	3.05 ± 1.43	< 0.001**	Normal-Osteopenia, Normal-Osteoporosis
Ba-C3ai (mm)	113.4 ± 8.3	112.0 ± 8.5	112.4 ± 9.1	0.694	
C0-C1 (mm)	12.0 ± 6.6	13.9 ± 5.9	10.4 ± 5.3	0.189	
**Lumbar spine**					
	Normal-L (n = 36)	Osteopenia-L (n = 53)	Osteoporosis-L (n = 27)	*P* value	
SNA (degree)	82.6 ± 3.2	82.4 ± 4.0	82.4 ± 3.8	0.958	-
SNB (degree)	78.8 ± 3.5	78.5 ± 3.3	78.2 ± 4.4	0.848	-
ANB (degree)	3.82 ± 2.31	3.86 ± 2.67	4.13 ± 2.28	0.876	-
NSL-OPT (degree)	79.9 ± 6.3	77.6 ± 6.1	79.4 ± 8.1	0.255	-
NSL-CVT (degree)	73.2 ± 5.7	72.3 ± 5.6	74.2 ± 8.3	0.452	-
OPT-CVT (degree)	6.67 ± 2.36	5.46 ± 2.65	5.26 ± 3.24	0.043*	-
Ba-C3ai (mm)	113.8 ± 9.3	114.1 ± 8.1	108.2 ± 5.8	0.007*	Normal-Osteoporosis
C0-C1 (mm)	12.7 ± 6.8	13.6 ± 6.0	11.4 ± 6.0	0.325	-

NSL, nasion-sella line; OPT, posterior tangent to the odontoid process through inferior posterior point of C2; CVT, posterior tangent to the odontoid process through inferior posterior point of C4; Ba-C3ai, the distance between basion (Ba) and the most inferior-anterior point on the body of the third vertebra (C3ia); C0-C1, the distance between base of the occiput and the posterior arch of the atlas.

Descriptive values are shown as mean ± SD.

Data obtained from one-way ANOVA.

* P < 0.05,

** *P* < 0.001 by one-way ANOVA followed by Bonferroni’s post-hoc analysis.

### Relationships among BMD, cervical spine degeneration, neck pain, and head and neck posture

The significant association between aBMD of the total hip and femoral neck and degree of osteophyte formation at the level of C2-3 and disc height loss at the level of C4-5 was observed. On the other hand, aBMD of the lumbar spine showed significant interactions with the extent of the disc height loss at the level of C4-5 only. There was a significant relationship between the degree of subjective neck pain and OPT-CVT with aBMD of the total hip, femoral neck, and lumbar spine (Table 4).

**Table 4:** Multivariate linear regression analysis for the bone mineral density.

**aBMD of total hip**				
R^2^ = 0.392 *P* < 0.001**	B	*P* value	95% CI	
Age	0.001	0.952	-0.003	0.003
BMI	0.007	0.055	0	0.013
C2-3 height loss 1	Reference			
C2-3 height loss 2	0.011	0.655	-0.039	0.062
C2-3 height loss 3	0.012	0.700	-0.051	0.076
C2-3 osteophyte formation 1	Reference			
C2-3 osteophyte formation 2	0.070	0.007*	0.020	0.121
C2-3 sclerosis 1	Reference			
C2-3 sclerosis 2	-0.044	0.095	-0.096	0.008
C2-3 sclerosis 3	0.031	0.466	-0.053	0.114
C3-4 height loss 1	Reference			
C3-4 height loss 2	-0.032	0.295	-0.092	0.028
C3-4 height loss 3	-0.023	0.456	-0.086	0.039
C3-4 osteophyte formation 1	Reference			
C3-4 osteophyte formation 2	0.009	0.715	-0.042	0.060
C3-4 sclerosis 1	Reference			
C3-4 sclerosis 2	-0.058	0.074	-0.123	0.006
C3-4 sclerosis 3	-0.001	0.965	-0.060	0.058
C4-5 height loss 1	Reference			
C4-5 height loss 2	-0.007	0.881	-0.098	0.085
C4-5 height loss 3	0.057	0.041*	-0.112	-0.002
C4-5 osteophyte formation 1	Reference			
C4-5 osteophyte formation 2	-0.018	0.562	-0.078	0.043
C4-5 sclerosis 1	Reference			
C4-5 sclerosis 2	0.003	0.977	-0.201	0.207
C4-5 sclerosis 3	-0.010	0.923	-0.212	0.192
NDI	0.005	0.011*	-0.010	0.001
OPT-CVT (degree)	0.017	< 0.001**	-0.025	-0.009
**aBMD of femoral neck**				
R^2^ = 0.302 *P* < 0.001**	B	*P* value	95% CI	
Age	0.001	0.770	-0.003	0.003
BMI	0.003	0.304	-0.003	0.010
C2-3 height loss 1	Reference			
C2-3 height loss 2	0.017	0.500	-0.033	0.067
C2-3 height loss 3	0.038	0.234	-0.025	0.102
C2-3 osteophyte formation 1	Reference			
C2-3 osteophyte formation 2	0.082	0.002*	0.032	0.133
C2-3 sclerosis 1	Reference			
C2-3 sclerosis 2	-0.035	0.178	-0.087	0.016
C2-3 sclerosis 3	-0.003	0.949	-0.086	0.081
C3-4 height loss 1	Reference			
C3-4 height loss 2	-0.034	0.257	-0.094	0.026
C3-4 height loss 3	-0.053	0.095	-0.115	0.009
C3-4 osteophyte formation 1	Reference			
C3-4 osteophyte formation 2	-0.001	0.999	-0.051	0.051
C3-4 sclerosis 1	Reference			
C3-4 sclerosis 2	-0.048	0.143	-0.112	0.017
C3-4 sclerosis 3	0.012	0.684	-0.047	0.071
C4-5 height loss 1	Reference			
C4-5 height loss 2	-0.028	0.548	-0.118	0.063
C4-5 height loss 3	-0.058	0.039*	-0.112	-0.003
C4-5 osteophyte formation 1	Reference			
C4-5 osteophyte formation 2	-0.017	0.573	-0.077	0.043
C4-5 sclerosis 1	Reference			
C4-5 sclerosis 2	0.121	0.099	-0.023	0.265
C4-5 sclerosis 3	0.001	0.966	-0.047	0.049
NDI	-0.003	0.045*	-0.006	0
OPT-CVT (degree)	-0.012	0.003*	-0.020	-0.004
**aBMD of total lumbar spine**				
R^2^ = 0.312 *P* < 0.001**	B	*P* value	95% CI	
Age	0.004	0.169	-0.002	0.009
BMI	0.004	0.169	-0.008	0.016
C2-3 height loss 1	Reference			
C2-3 height loss 2	-0.005	0.909	-0.095	0.085
C2-3 height loss 3	-0.038	0.489	-0.149	0.072
C2-3 osteophyte formation 1	Reference			
C2-3 osteophyte formation 2	0.009	0.838	-0.079	0.098
C2-3 sclerosis 1	Reference			
C2-3 sclerosis 2	0.004	0.936	-0.088	0.095
C2-3 sclerosis 3	0.106	0.159	-0.043	0.254
C3-4 height loss 1	Reference			
C3-4 height loss 2	0.042	0.566	-0.105	0.190
C3-4 height loss 3	0.025	0.654	-0.086	0.137
C3-4 osteophyte formation 1	Reference			
C3-4 osteophyte formation 2	0.016	0.727	-0.075	0.107
C3-4 sclerosis 1	Reference			
C3-4 sclerosis 2	-0.017	0.813	-0.156	0.123
C3-4 sclerosis 3	-0.014	0.790	-0.119	0.091
C4-5 height loss 1	Reference			
C4-5 height loss 2	0.022	0.754	-0.116	0.160
C4-5 height loss 3	0.099	0.043*	0.003	0.195
C4-5 osteophyte formation 1	Reference			
C4-5 osteophyte formation 2	-0.002	0.969	-0.110	0.105
C4-5 sclerosis 1	Reference			
C4-5 sclerosis 2	-0.178	0.332	-0.542	0.186
C4-5 sclerosis 3	-0.218	0.231	-0.578	0.142
NDI	0.007	0.013*	-0.013	-0.002
OPT-CVT (degree)	0.015	0.036*	-0.030	-0.001

BMI, body mass index; C2, the second vertebra; C3, the third vertebra; C4, the forth vertebra; C5, the fifth vertebra; 1, mild degeneration; 2, moderate degeneration; 3, severe degeneration; VAS, visual analog scale; NDI, neck disability index; OPT, posterior tangent to the odontoid process through inferior posterior point of C2; CVT, posterior tangent to the odontoid process through inferior posterior point of C4.

Data obtained from the multivariate linear regression.

* P < 0.05,

** *P* < 0.001 by the multivariate linear regression.

## Discussion

The associations among the degenerative changes of the cervical spine, head and neck postures, and neck pain in elder population have been investigated, previously [3]. Many studies have been undertaken to reveal the interactions between BMD of the lumbar and hip and degree of lumbar spine degeneration [9–13,26,27]. Furthermore, the degree of lumbar disc degeneration seemed to have interactions with altered lumbar lordosis and kinematics [8]. The reduced paraspinal muscle volume and strength due to aging seemed to have impact on decreased stabilization of the vertebral spine and abnormal force transmission [1,2,4]. However, sparse reports ever have focused on the associations between the cervical spine degeneration and BMD. Hence, the purpose of the present study was to clarify the relationship between degenerative changes in the cervical spine, head and neck postures, neck pain, and BMD of the total hip and lumbar spine in the elder females.

Aforementioned results exhibited a significant interaction between degenerative changes of the cervical spine and osteoporosis. Several previous studies focused on the pathophysiological interactions between the disc degeneration of the vertebrae and osteoporosis such as negative impact of osteoporosis on the disc degeneration in the lumbar spine due to failure of adequate supplement of nutrient to the disc cell [28,29] and increased endplate thinning and microfracture owing to poor bone quality [30]. Even clinical studies demonstrated that lower lumbar BMD was associated with a decreased lumbar disc volume and altered spinal kinematics [31,32]. However, studies involving middle-aged subjects, less than 64-year-old showed opposite results that individuals with more advanced degenerative changes in lumbar spine tended to have higher BMD in the lumbar spine and hip [26,27]. A commentary suggested one hypothesis that in middle-aged subjects with high levels of peak spinal loading and bone strength, dense vertebral bone could possibly threaten the adjacent discs by increasing pressure in the disc nucleus [33]. The average age of the participants of the present study was 60.4 ± 7.1 years old and this could explain the positive relationship between higher BMD of the lumbar spine and hip, and advanced cervical spine degeneration.

The results from multivariate linear regression analysis demonstrated a strong association between the extent of disc degeneration at the lower cervical spine, and BMD of the lumbar spine and hip. The segment-specific effects, more compressive mechanical loading and a different strain distribution model could be assumed in lower level of the spine [34]. In addition, a stiffened vertebrae could cause elevated mechanical loading on the adjacent disc, whereas an osteoporotic vertebrae might cushion or protect the disc from degeneration [35]. Hence, lower BMD of the vertebrae possibly reduce the mechanical stress to the disc of the cervical spine and due to higher mechanical load from the weight to the lower cervical spines compared to upper ones and the interaction between disc degeneration and BMD was more prominent at the lower levels of cervical spine.

The novel finding from this study was positive associations between FHP, neck pain, and BMD of the lumbar spine and hip. Advanced cervical spine degeneration may accompany altered head position and neck pain [3]. Furthermore, individuals with higher BMI demonstrated more FHP due to compensatory effort against a compromised airway volume owing to fat deposition [36]. Participants with greater BMD exhibited higher BMI and this might have an association with FHP. Therefore, we carefully suggested one hypothesis that higher BMI from females with higher BMD could have a role in the development of FHP, cervical spine degeneration, and neck pain.

The present study adopted indices from lateral cephalogram that disc degeneration was assessed by the extent of vertebral height loss. However, osteoporosis seemed to be associated with vertebral body height loss, which could allow for vertical expansion of the disc [31]. Therefore, the osteoporotic spine might show less degree of disc space narrowing, and less likely be graded as a disc degeneration.

To the best of our knowledge, this is the first attempt to reveal the association between the cervical spine degeneration, head and neck postures, neck pain, and BMD of the lumbar spine and hip in post-menopausal females. However, we encountered several limitations. Firstly, including only female patients gave limited information. Due to the characteristics of the retrospective study design and female preponderance in the TMD and osteoporosis, enrollment of the sufficient number of males was very difficult. Moreover, sufficient evaluation tool for evaluation of cervical spine disorders such as magnetic resonance imaging were not available. Secondly, because all participants in the present study were patients with TMD, the extent of the orofacial pain could influence the head posture and neck pain. To overcome this limitation, homogeneity of the samples in each group including parameters related with TMD was confirmed through statistical analysis. Therefore, this could not critically lessen the value of this study. Thirdly, because cephalograms could not cover the lower portion of the cervical spine, the information about angulation and alignment of the fifth, sixth, and seventh cervical vertebra could not be derived from the present study. Fourthly, the causality among those factors could not be derived from this study owing to retrospective study design. Future prospective study with larger samples including both males and females would be needed.

In conclusion, this cross-sectional study demonstrated that post-menopausal females with higher BMD exhibited more FHP, higher levels of neck pain, and greater degree of the cervical spine degeneration. Many contributing factors such as compressive mechanical loading, lower BMI, and methodology of analyzing degree of the cervical spine degeneration could have impact on those findings. A compressive understanding of the association between BMD, cervical spine degeneration, head and neck posture, and neck pain could possibly lead to novel insights into the interaction between vertebrae and disc and the etiology of cervical spine degeneration and neck pain.
